# Analyzing the Relationship between Dose and Geometric Agreement Metrics for Auto-Contouring in Head and Neck Normal Tissues

**DOI:** 10.3390/diagnostics14151632

**Published:** 2024-07-29

**Authors:** Barbara Marquez, Zachary T. Wooten, Ramon M. Salazar, Christine B. Peterson, David T. Fuentes, T. J. Whitaker, Anuja Jhingran, Julianne Pollard-Larkin, Surendra Prajapati, Beth Beadle, Carlos E. Cardenas, Tucker J. Netherton, Laurence E. Court

**Affiliations:** 1 Department of Radiation Physics, The University of Texas MD Anderson Cancer Center, Houston, TX 77030, USA; rmsalazar1@mdanderson.org (R.M.S.); tjwhitaker@mdanderson.org (T.J.W.); jmpollard@mdanderson.org (J.P.-L.); lecourt@mdanderson.org (L.E.C.); 2 The University of Texas MD Anderson Cancer Center UTHealth Houston Graduate School of Biomedical Sciences, Houston, TX 77030, USA; 3 Department of Statistics, Rice University, Houston, TX 77005, USA; ztw5@rice.edu; 4 Department of Biostatistics, The University of Texas MD Anderson Cancer Center, Houston, TX 77030, USA; 5 Department of Imaging Physics, The University of Texas MD Anderson Cancer Center, Houston, TX 77030, USA; 6 Department of Radiation Oncology, The University of Texas MD Anderson Cancer Center, Houston, TX 77030, USA; 7 Department of Radiation Oncology–Radiation Therapy, Stanford University, Stanford, CA 94305, USA; bbeadle@stanford.edu; 8 Department of Radiation Oncology, The University of Alabama, Birmingham, AL 35294, USA

**Keywords:** auto-contouring, contouring, radiotherapy, organs-at-risk, head and neck

## Abstract

This study aimed to determine the relationship between geometric and dosimetric agreement metrics in head and neck (H&N) cancer radiotherapy plans. A total 287 plans were retrospectively analyzed, comparing auto-contoured and clinically used contours using a Dice similarity coefficient (DSC), surface DSC (sDSC), and Hausdorff distance (HD). Organs-at-risk (OARs) with ≥200 cGy dose differences from the clinical contour in terms of D_max_ (D0.01cc) and D_mean_ were further examined against proximity to the planning target volume (PTV). A secondary set of 91 plans from multiple institutions validated these findings. For 4995 contour pairs across 19 OARs, 90% had a DSC, sDSC, and HD of at least 0.75, 0.86, and less than 7.65 mm, respectively. Dosimetrically, the absolute difference between the two contour sets was <200 cGy for 95% of OARs in terms of D_max_ and 96% in terms of D_mean_. In total, 97% of OARs exhibiting significant dose differences between the clinically edited contour and auto-contour were within 2.5 cm PTV regardless of geometric agreement. There was an approximately linear trend between geometric agreement and identifying at least 200 cGy dose differences, with higher geometric agreement corresponding to a lower fraction of cases being identified. Analysis of the secondary dataset validated these findings. Geometric indices are approximate indicators of contour quality and identify contours exhibiting significant dosimetric discordance. For a small subset of OARs within 2.5 cm of the PTV, geometric agreement metrics can be misleading in terms of contour quality.

## 1. Introduction

Contouring accuracy in radiotherapy is important for achieving optimal outcomes. This is especially true in head and neck (H&N) cancer treatment, where the therapeutic window is narrow due to the proximity of the planning target volume (PTV) to organs-at-risk (OARs) [1,2,3]. Advances in auto-contouring are reducing inconsistencies inherent in manual delineation of PTVs and OARs and making this time-consuming, experience-dependent skill more efficient [4,5,6]. Researchers predominantly use geometric agreement metrics to assess the potential clinical impact of auto-contouring tools for deployment in radiotherapy planning or contour quality assurance [7,8,9,10]. Specifically, in 99.1% of studies presenting a new or previously established auto-contouring model, researchers reported geometric agreement metrics like similarity and overlap of a ground truth and auto-generated prediction. Conversely, in just 23.1% of studies, researchers reported the dosimetric impact [7].

Although frequently used, researchers have yet to show that geometric agreement metrics meaningfully correlate with the clinical acceptability of auto-contours or the potential implications regarding the final treatment dosimetry [11,12,13]. Some studies have considered the effect of geometric variations in contours on plan dose distributions. Fung et al. found that, despite generally small geometric discrepancies between manual- and auto-contours generated for OARs in adaptive radiotherapy for nasopharyngeal carcinoma, auto-contours often resulted in statistically significant higher doses in critical areas [14]. They suggest that even small geometric discrepancies can have significant dosimetric impacts due to steep dose gradients typical in IMRT; thus, auto-contours should be manually adjusted to ensure treatment safety and efficacy. Conversely, van Rooij et al. observed that even lower Dice similarity coefficients between deep learning-based and manual contours still produced dosimetric differences in treatment plans that were often clinically irrelevant; in this context, the study supports clinical acceptability of some automated delineations despite geometric inaccuracies [15]. In magnetic-resonance-imaging-based OAR auto-contours for brain tumor patients, Turcas et al. also found that geometric variations had minimal impact on dose distribution versus manual contours, where differences in median values for the mean and max dose were less than 0.2 Gy [16]. Notably, Lim et al. found that the correlation between geometric and dosimetric agreement metrics was weak (R2 < 0.2 for 61% of the correlations studied) and inconsistent when looking at plans generated from different combinations of physician expert-drawn target and OAR contours and resident physician-drawn contours over nasopharyngeal cancer cases [17]. They concluded that a dosimetric effect owing to contouring variation is not significantly captured with geometric indices alone.

Given these challenges, this study sought to further clarify the link between geometric agreement metrics commonly used in evaluating auto-contouring and dosimetric differences in H&N OARs. This study quantifies the impact of geometric edits on auto-contours in terms of the mean and maximum dose extracted from clinically delivered treatment plans.

## 2. Materials and Methods

Patients received H&N photon volumetric modulated arc radiotherapy (VMAT) plans designed with computed tomography (CT)-based manually drawn target contours and auto-generated OAR contours that were then manually edited. The dose distribution of the clinical plan was applied over the original, un-edited set of auto-contour OARs; dose statistics were compared between the auto-contour and the clinical OAR.

### 2.1. Contours and Plan Dose

Two H&N OAR contour sets were evaluated in this study: auto-contours and clinical contours. The auto-contour OARs were generated by the Radiation Planning Assistant (RPA), a full-automated treatment planning system whose deep-learning contouring is used routinely for delineating H&N OARs at our institution [18,19]. Clinical contours refer to the OAR contours that were automatically generated, then reviewed and modified by clinical planners prior to use for treatment planning. In this way, an “automated” set and a “clinical” set of contours were compared. In total, the following 19 OARs were evaluated: brain, brainstem, cochleas, esophagus, eyes, submandibular glands, larynx, lens, optic chiasm, optic nerves, parotids, spinal cord, and vertebral column.

The clinical VMAT radiotherapy plan–generated with the manually drawn target volume contours and the clinical (auto-generated, manually edited) OAR contours–was retrospectively collected. Then, the resulting doses to the original, unedited auto-contours and the clinical contours were extracted to compare dose statistics between the two contour sets.

### 2.2. Dataset

This study utilized a dataset of 287 patients from our institution to conduct an initial analysis; then, an external multi-institutional set of 91 patients were evaluated to validate the findings.

Internal. Radiotherapy treatment plan data from 287 H&N cancer patients treated with VMAT between August 2020 and February 2023 at our institution were retrospectively collected for this study. The planned doses ranged from 14 to 70 Gy, with 1–3 dose levels over 5–35 fractions; all patients received at least 2 Gy per fraction. Patients had RPA auto-contours available during contouring and were planned on a Raystation treatment planning system (version 11B, RaySearch Laboratories, Stockholm, Sweden).

External. Radiotherapy treatment plan data from 91 H&N cancer patients treated with VMAT between January 2022–November 2022 across 4 institutions in the continental United States were retrospectively collected. Planned total doses for these patients ranged from 60 to 70 Gy, with 1–3 dose levels over 25–35 fractions; all patients received at least 2 Gy per fraction.

### 2.3. Geometric Indices

The H&N OAR auto-contours were compared to the clinical contours using the Dice similarity coefficient (DSC), surface Dice similarity coefficient (sDSC), and Hausdorff distance (HD). These metrics were selected owing to their widespread reporting and demonstrated effectiveness in detecting discrepancies in independently generated auto-contours [7,9,12,20]. For an sDSC, we chose a tolerance of 2 mm as the center value of previously studied tolerance levels with a similar accuracy in detecting contouring errors [9]. Additionally, distance to the closest PTV, defined by the Euclidean distance between the OAR contour and PTV, was collected. The previous literature has suggested that the relation between dose differences and shortest distance to the edge of PTV provides a robust tolerance guideline for contouring variability and calls for a larger dataset to determine a distance cut-off point with more detail [21,22,23].

### 2.4. Dosimetric Indices

The clinical plan dose was applied over the two contour sets being compared in this study. The established clinical objective criteria for H&N cancer treatment plans at our institution specify constraints in terms of D_max_ (as defined by the maximum dose received by at least 0.01 cc of the volume) for the brain, brainstem, cochlea, esophagus, eyes, lens, optic chiasm, optic nerves, spinal cord, vertebral column and D_mean_ for larynx, submandibular glands, and parotids. The clinical objective dose (D_CO_) for each respective organ is reported and analyzed as specified (D_max_ for most organs, D_mean_ for larynx, submandibular glands, and parotids).

### 2.5. Statistical Analysis

Effect of contour variations on dose differences. The mean and standard deviation of geometric similarity between the clinical OARs auto-contour OARs was reported in terms of DSC, sDSC, and HD. The difference in D_max_ and D_mean_ doses between the two contour sets was tested for statistical significance using a 2-sided Wilcoxon signed-rank test (*p* < 0.05). Then, the relationship between geometric and dosimetric indices was evaluated with the absolute value of dosimetric differences; thus, the median and 90% quantile are reported to describe the spread of observed differences in D_max_ and D_mean_.

### 2.6. Linear Relationship

It was investigated whether a linear relationship between geometric and dosimetric indices existed for the entire dataset and for each individual organ. The R2 statistic was used to determine the strength of correlation.

### 2.7. Thresholding Dose Differences and Distance to the PTV

This study identified contours with a threshold of a 200 cGy absolute difference in dose, with respect to the clinical objective dose metric D_CO_ for each organ, between the two contour sets. This was used to further analyze characteristics of dosimetric deviation in terms of geometric similarity and distance to the closest PTV in a subset of contours. This criterion was chosen as 200 cGy is about 3%, or 1 fraction, of the total target dose prescription typical of an H&N radiotherapy treatment plan (69.96 Gy/33 fractions). All patients in the internal dataset who had a wide planned dose inclusion range received at least 200 cGy per fraction. The distance to PTV is investigated as a potential metric to identify contour pairs who may exhibit high geometric similarity but low dosimetric similarity and vice versa. Illustrative cases are provided for both scenarios. All calculations were made using custom Python scripts that leverage common scientific libraries.

To validate the findings for our internal dataset and assess the generalizability of the observed trends, an external, multi-institutional dataset for 91 H&N cancer patients previously treated with radiotherapy were also evaluated with the same methodology. The purpose of this extended analysis was to ascertain whether the patterns and discrepancies identified in the original cohort were consistent in a broader clinical context.

## 3. Results

### 3.1. Internal Dataset–Geometric and Dose Evaluation

The 287 internal patient dataset each had plans containing up to 13 unique OAR contour pairs for a total of 4995 comparison pairs (original auto-contour vs. clinical contour). Although we evaluated 287 patients, the sample size per OAR did not homogenously total 287 because clinicians delete certain contours deemed nonrelevant for a specific patient’s radiotherapy plan (or otherwise the organ was part of the target volume). Treatment site characteristics for the 287 patients are displayed in Table 1.

Summary statistics for geometric and dosimetric agreement metrics can be found in Table 2. ΔDose was calculated and reported as the absolute value of the auto-contour dose minus the clinical contour dose. Overall, 90% of contours had a DSC, sDSC, and HD of at least 0.75, 0.86, and less than 7.65 mm, respectively. Notably, the esophagus, larynx and spinal cord exhibited high geometric variation in the summary statistics. Though 254/282 spinal cords exhibited a HD of <4 mm, 11 cases exhibited >40 mm and thus affected the observed standard deviation (though these cases did not correlate with differences to D_max_, where only 2/11 cases exhibited more than 200 cGy difference). In general, these organs exhibited such variations due to the clinical planners removing contours from slices distant from the treatment volume (where the auto-contour delineates the full anatomical extent of the organ).

Dosimetrically, the absolute difference between the two contour sets was less than 200 cGy for 95% of OARs in terms of D_max_ and 96% in terms of D_mean_. The clinical objective criteria at our institution specify constraints in terms of D_max_ (as defined by the maximum dose inside a volume of 0.01 cc) for most OARs and D_mean_ for the larynx, submandibular glands, and parotids; thus, in terms of their clinical objective dose D_CO_, 96% of relevant organs had D_max_ < 200 cGy and 90% had D_mean_ < 200 cGy. The signed differences in dose between the two contour sets was not found to be statistically significant (*p* = 0.34, 0.45 for D_max_, D_mean_).

### 3.2. Internal Dataset–Linear Relationship

Overall, the linear relationship between geometric agreement metrics and the dose difference when considering all contours was poor. For N = 4995, the R2 between D_max_ and the DSC, sDSC, and HD were 0.09, 0.14, and 0.04, respectively. This is likely due to 57% (2874/4995) of contour pairs exhibiting a 0 dose difference (the auto-contour OAR was used as-is). However, re-assessing the linear relationship, thresholding for a minimum D_max_ difference of 100 cGy across all contours, does not improve the correlation (R2 = 0.06, 0.06, and 0.01, respectively).

**Figure 1:**
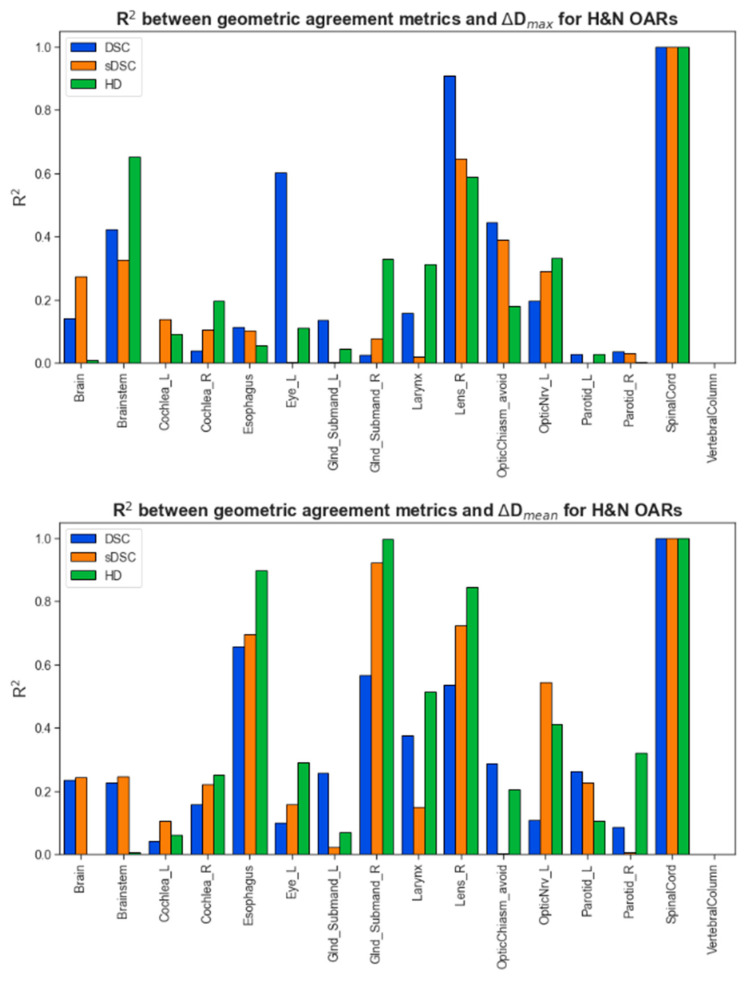
The correlation between geometric and dosimetric incides from clinical contours vs. auto-contours, as compared over one clinical plan dose distribution.

Figure 1 summarizes the correlation between geometric and dosimetric agreement metrics by OAR. Fitting a model to each organ improved the correlation strength, though no geometric index showed a consistently better performance over most OARs. Generally, geometric agreement metrics correlated more strongly with ΔD_mean_ than ΔD_max_. Expectedly, DSC showed a stronger correlation with ΔD_mean_, where it exhibited a markedly varied performance with ΔD_max_. This is exhibited later when examining cases that scored an excellent DSC yet poor dosimetric agreement in terms of ΔD_max_, highlighting the nuances of capturing errors with volumetric overlap in large versus smaller structures.

### 3.3. Thresholding Differences and Distance to the PTV

A threshold of 200 cGy absolute dose difference in D_CO_ resulted in 229/4995 (4.5%) of the total structures being identified. Table 2 summarizes the sample size of each OAR that fit the criterion, the clinical objective dose metric considered, the relative number of OARs identified against their sample size in the total dataset, the number of OARs within 3 cm of the PTV, and the observed range of dose variance in terms of D_CO_.

**Figure 2:**
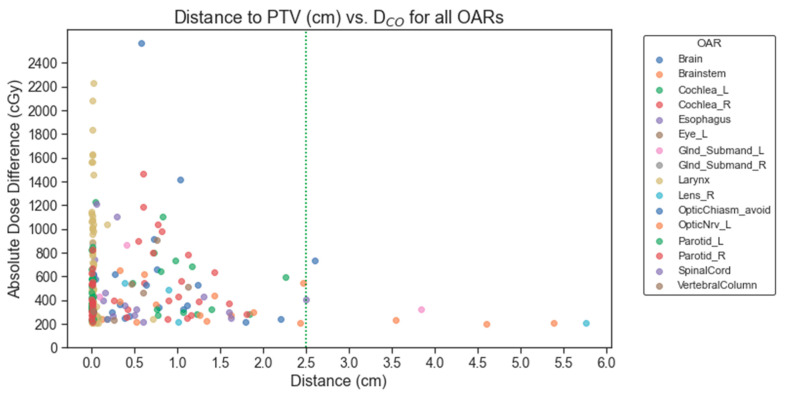
The relationship between identifying an OAR for exhibiting ΔD_CO_ ≥ 200 cGy deviation between the clinical contour and the auto-contour and relative distance to the closest PTV.

Notably, 97% (223/229) of OARs meeting this criterion were within 2.5 cm of the nearest PTV; thus, the distance-to-nearest-PTV metric exhibited a markedly higher sensitivity to identifying cases where two contours may disagree dosimetrically (though not necessarily geometrically). For example, 27% (60/224) OARs with ΔD_CO_ had DSC > 0.90; all 60 were within 2.5 cm of the PTV. Additionally, 31% (70/224) had sDSC > 0.90 or HD < 5 mm, of wfhich 68 were within 2.5 cm of the PTV (and 2 were beyond 5 cm). These findings are summarized in Figure 2.

**Figure 3:**
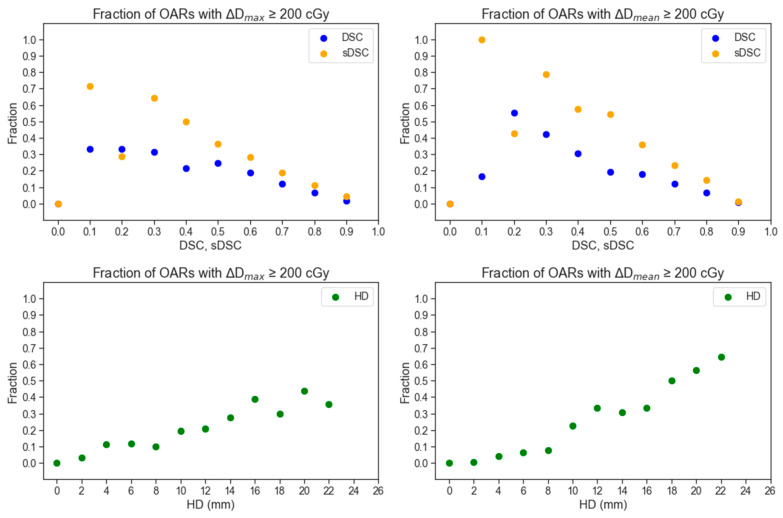
The relative proportions of contours across all OARs identified for exhibiting a dose deviation of at least 200 cGy in terms of D_max_ (D0.01 cc) or D_mean_ versus binned geometric scores. DSC and sDSC were binned at every 0.1 interval and HD was binned at every 2 mm.

**Figure 4:**
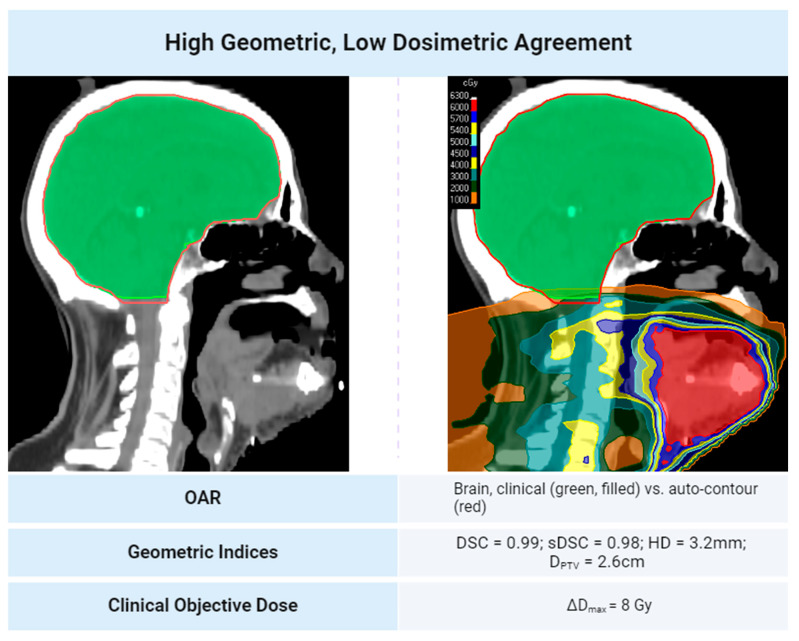
A treatment plan for a bilateral neck tumor for which a high geometric index score did not indicate high dosimetric agreement. The prediction difference in the inferior aspect of the brain for the auto-contour encompassed additional isodose regions that the clinical contour did not on some slices.

**Figure 5:**
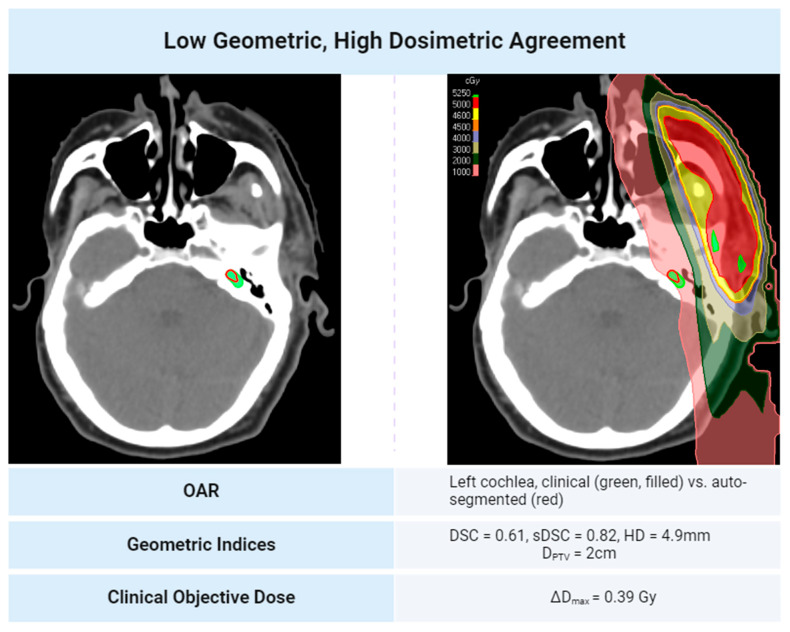
A treatment plan for a left cheek tumor for which a low geometric agreement did not indicate low dosimetric agreement. The two contours lie in an approximately homogenous isodose region 2 cm away from the PTV in the same laterality.

Generally, high geometric agreement reduces the chance of a 200 cGy dose discrepancy between two contour sets in H&N treatment plans. This relationship is shown in Figure 3. Some noise in the linear decrease between geometric agreement and dosimetric disagreement exists because most of the data collected is skewed towards higher geometric agreement scores and lower dosimetric disagreement. When OARs are within 2.5 cm of the PTV, the trend may not follow. This suggests that proximity to a clinical target heightens the dosimetric error potential, even with geometrically similar contours. Notably, OARs distant from the PTV, even those with low geometric agreement, do not consistently indicate significant dose discrepancies based on the clinical plan’s isodose lines. Cases illustrating the discrepancies in geometric and dosimetric agreement are shown in Figure 4 and Figure 5. A treatment plan for a bilateral neck tumor for which a high geometric agreement did not indicate high dosimetric agreement is shown in Figure 4. Although the auto-contour of the brain (red) had a DSC = 0.99, sDSC = 0.98, and HD = 3.2 mm against the clinical contour (green, filled), the two structures differed by 8 Gy reported to the D_max_. The prediction difference in the inferior aspect of the brain for the auto-contour encompassed additional isodose regions that the clinical contour did not on some slices. A treatment plan for a left cheek tumor for which a low geometric agreement did not indicate low dosimetric agreement is shown in Figure 5. Although the left cochlea auto-contour prediction (red) was substantially smaller than the clinical contour (green, filled), with a DSC = 0.61, sDSC = 0.82, and HD = 4.9 mm, the two contours were completely encompassed in a 10 Gy isodose region 2 cm away from the PTV in the same laterality. Thus, the D_max_ difference between the two cochleae was 31 cGy.

### 3.4. External Dataset

We repeated the workflow described above on a multi-institutional dataset of 91 patients, obtaining 1129 OAR comparison pairs. The mean (± SD) DSC, sDSC, and HD (mm) for all OARs in this dataset were 0.79 ± 0.19, 0.80 ± 0.21, and 12 ± 29, respectively. Again, the esophagus, larynx, and spinal cords exhibited high geometric variability between the clinical sub-volume being considered for planning and the auto-contour delineating the full anatomical extent. However, despite higher geometric discordance, the dosimetric agreement was within 200 cGy for 95% for contours overall in terms of D_max_ and D_mean_ and 94% and 90% for contours in terms of their D_CO_ of D_max_ and D_mean_, respectively. Correlation strength between geometric and dosimetric agreement metrics were markedly poor for the external dataset (R2 < 0.40 for all correlations except for spinal cord and vertebral column). Notably, 53/1129 (4%) OARs exhibited ΔD_CO_ ≥ 200 cGy, of which 32/53 (60%) were within 2.5 cm of the PTV.

## 4. Discussion

In this work, we found that geometric agreement metrics commonly used to compare contours are approximate indicators of quality. As the geometric disagreement increases, there is an increased likelihood of a meaningful dosimetric difference between two contours. Notably, this study reported on the relationship between geometric agreement and dosimetric endpoints in auto-contours that are being used in a clinical context, providing insight on the clinical acceptability (readiness of use) with metrics instead of qualitative evaluation (physician scoring). Variations in contouring when using auto-contours as a starting point were significantly low (with most contours being used as-is). This study transposed auto-contours on the clinical treatment plan as an approximate indicator of how geometric edits might affect final plan dose to enable a study of a large patient cohort compared to replanning studies.

We were able to investigate 13 key OARs that are routinely contoured in our clinical practice; however, additional organs and muscles, which may be delineated in other planning practices, should also be assessed. Notably, the swallowing organs may be of interest, as they are associated with dysphagia, aspiration, and general quality of life. While not analyzed in this study, we can infer similar effects of contouring differences on dose to these swallowing structures due to the anatomical proximity and volumetric similarity to organs in our study. The OARs analyzed in this work cover a wider range of volumes and proximities to the treatment targets. These include small organs such as the lenses (average volume of 0.20 cm^3^) and optic nerves (0.72 cm^3^), medium-sized organs like the spinal cord (22 cm^3^) and brainstem (26 cm^3^), and large organs such as the brain (1382 cm^3^). Swallowing organs tend to have similar volumes to the small and medium OARs included in this study. For instance, the buccinator muscles (5 cm^3^) are comparable in size to the submandibular glands (8 cm^3^) and are also in close proximity. Furthermore, some of these swallowing muscles are smaller, anatomically continuous volumes of analyzed organs, like the esophagus and pharyngeal constrictor muscles or the thyroid and cricoid cartilage and the larynx. A volumetric comparison of 23 swallowing organs to the 13 key OARs analyzed in this study can be found in the Appendix A.

Additionally, due to the inclusion of diverse prescriptions and fractionations in the initial dataset, we conducted additional analyses to address potential discrepancies in the results. We modified the inclusion criteria to exclusively consider patients who received at least 40 Gy in their treatment and identified structures based on dosimetric discrepancies as a percentage of the total plan dose (rather than absolute dose), considering both 3% and 5% of the prescribed dose. Changing the inclusion criteria in terms of plan dose and identification threshold in terms of a percentage of the prescribed dose did not change the conclusions drawn in this study; the same trends were observed as summarized in Figure 2 and Figure 3. This consideration pertains to VMAT plans, where contours and prescriptions prepared for other radiotherapy treatment modalities, such as proton therapy, should also be evaluated.

This study also highlighted some flaws in the use of geometric agreement metrics to determine the clinical readiness of auto-contours. Specifically, even if the geometric index indicates high agreement, there may still be large dosimetric differences when the OAR is close to target structures. Flaws in the use of geometric metrics have been highlighted by other authors [24,25,26,27,28]. For example, a DSC can be lenient of contouring errors for large structures like the brain and parotids while penalizing errors in smaller structures like the eyes, cochleas, lenses, and optic nerves. Surface DSC tends to overlook the overlap between structures, which would be particularly challenging for evaluating OARS who overlap with the PTV and thus are at risk of having high dose differences between two contours. Additionally, an sDSC has differential sensitivity, with the ability to tune its tolerance value to surface errors when calculating. This study’s limitation was the application of a uniform tolerance of 2 mm to decrease variability in results. Such uniformity is not always suitable when studying structures that vary in size. The effects of structure size on calculating a Hausdorff distance are markedly lower. However, deletion of slices, a common contouring error (where the clinical planner forgets to interpolate manual contours) may not be identified by some of these geometric metrics. Again, these highlight the fact that these metrics should not be used without additional checks.

Our results indicated that additional review is critical for structures that are within 2.5 cm from the target. Specific tolerance levels of contouring variability against OAR distance-to-PTV have been previously studied and seen to be especially important for D_max_. Vaassen et al. suggested that a prescribed dose will have an impact on the proposed distance-to-PTV cut-off point for checking contours, but in our study, with almost 5000 contours and a wide range of dose levels, we found the significance of checking close structures at multiple prescriptions [21]. Further work of interest would include designing a novel metric that can account for geometric agreement and relative distance to a target volume.

The weaknesses identified in this study are caused by the fact that contouring quality is generally evaluated before the generation of a treatment plan, so the impact of contouring discrepancies on dose is unknown (at that point). The more recent introduction of deep learning to predict what the planned dose distribution will look like [29,30] means that it is possible now to compare contours and the dosimetric impact of any differences before the creation of a treatment plan. Given the findings of our work, especially the gaps that werre left when using overlap indices alone, it seems likely that future evaluation of clinical readiness of auto-contours will include dose predictions so that they can highlight errors based on dosimetric differences.

## 5. Conclusions

Our investigation showed that the relationship between geometric metrics and the dosimetric impact of differences in contours is not straightforward; thus, this should be considered when developing tools to support clinical acceptability of auto-contouring. We found that dose differences between an auto-contour and a clinical contour are small when using auto-contours as a starting point. Dose variances resulting from contour edits correlated moderately with geometric agreement metrics. Notably, the distance from an OAR to the PTV stood out as an essential metric for identifying OARs with geometric agreements but dosimetric divergences. By corroborating our primary dataset’s results with data from multiple institutions, we demonstrated the replicability and applicability of our primary findings across diverse datasets.

## Figures and Tables

**Table 1:** Treatment characteristics of the internal patient dataset from the Internal Classification of Diseases (ICD) coding.

Primary Site	Patients, No.
Bone & Soft Tissue	12
Larynx	19
Lip and Oral Cavity	54
Nasal Cavity	13
Oropharynx	104
Other	53
Skin	20
Thyroid	12
Clinical characteristics of the study patients (n = 287)	

**Table 2:** Summary statistics for geometric and dosimetric agreement for all organs. ΔDose = clinical contour dose–auto-contour dose. Then, a summary of OARs identified for exhibiting a ≥200 cGy dose deviation from the pertinent clinical objective between the clinical contour and the auto-contour.

														When D_CO_ ≥ 200 cGy		
OAR	*N*	DSC		sDSC		HD (mm)		ΔD_max_ (cGy)		ΔD_mean_ (cGy)		D_CO_ ≥ 200 cGy		*N* (% of total OARs)	*N* (% of OARs ≤ 3 cm from PTV)	Range ΔD_CO_ (cGy)
		Mean	SD	Mean	SD	Mean	SD	Median	Q90%	Median	Q90%	Max	Mean			
All structures	4995	0.90	0.11	0.95	0.1	3.91	9.22	0	55	0.67	49			229 (5%)	224 (97%)	201–2565
Brain	240	0.99	0.01	0.99	0.03	3.74	2.80	0	113	0	4	X		13 (5%)	13 (100%)	214–914
Brainstem	285	0.96	0.04	0.97	0.07	2.56	2.45	0	38	1	36	X		6 (2%)	6 (100%)	206–541
Cochlea_L	275	0.78	0.17	0.92	0.12	2.75	1.92	0	80	2	76	X		17 (6%)	17 (100%)	240–1225
Cochlea_R	278	0.81	0.14	0.95	0.08	2.41	1.45	0	130	2	62	X		20 (7%)	20 (100%)	238–1469
Esophagus	260	0.91	0.14	0.94	0.14	13.27	29.49	0	124	3	449	X		20 (8%)	20 (100%)	216–1213
Eye_L	271	0.96	0.04	0.99	0.05	1.82	1.42	0	24	0	6	X		6 (2%)	6 (100%)	234–905
Eye_R	273	0.96	0.03	0.99	0.03	1.7	0.84	0	12	0	4	X		0 (0%)	N/A	N/A
Glnd_Submand_L	238	0.91	0.12	0.94	0.13	3.36	3.94	0	36	1	51		X	7 (3%)	6 (86%)	248–863
Glnd_Submand_R	243	0.94	0.07	0.97	0.08	2.96	2.95	0	35	1	35		X	3 (1%)	3 (100%)	217–312
Larynx	245	0.87	0.15	0.81	0.23	7.83	7.39	2	512	26	799		X	68 (28%)	68 (100%)	204–2229
Lens_L	264	0.85	0.13	0.97	0.07	1.73	0.89	0	14	0	5	X		0 (0%)	N/A	N/A
Lens_R	268	0.86	0.1	0.98	0.05	1.74	0.80	0	10	0	5	X		4 (1%)	3 (75%)	211–544
OpticChiasm	209	0.93	0.12	0.97	0.1	1.75	1.74	0	5	0	6	X		6 (3%)	6 (100%)	266–2565
OpticNrv_L	269	0.83	0.12	0.95	0.08	4.28	4.57	0	52	1	49	X		12 (4%)	9 (75%)	201–654
OpticNrv_R	270	0.83	0.12	0.95	0.08	4.17	4.05	0	46	1	37	X		0 (0%)	N/A	N/A
Parotid_L	280	0.96	0.05	0.97	0.07	5.55	7.11	0	67	2	174		X	25 (9%)	25 (100%)	225–850
Parotid_R	282	0.96	0.03	0.97	0.06	4.68	5.47	0	46	2	107		X	19 (7%)	19 (100%)	213–820
SpinalCord	282	0.95	0.07	0.98	0.07	5.39	19.22	0	21	1	14	X		2 (0.01%)	2 (100%)	247–302
VertebralColumn	263	0.98	0.01	0.99	0.01	2.62	2.26	0	13	1	10	X		1 (0.5%)	1 (100%)	305

## Data Availability

The datasets presented in this article are not readily available because they are not anonymized patient data from our institution. Requests to access the datasets should be directed to Laurence E. Court.

## Appendix A

**OAR**	**Average Volume (cm^3^)**
Lens	0.20
Cochlea	0.22
Optic Nerve	0.72
Optic Chiasm	0.75
Medial Constrictor *	1.08
Glottic Area *	1.09
Cricopharyngeus *	2.03
Inferior Constrictor *	2.27
Digastric *	3.51
Buccinator *	5.07
Cricoid *	6.20
Lateral Pterygoid *	7.48
Eyes	7.94
Submandibular Glands	8.12
Medial Pterygoid *	9.75
Thyroid Cartilage *	9.74
Superior Constrictor *	12.60
MGH Complex *	13.85
Supraglottic Larynx *	14.49
Esophagus	21.16
SpinalCord	22.87
Genioglossus *	23.35
Masseter *	24.70
Brainstem	26.55
Parotid	33.41
Larynx	36.33
Tongue *	41.25
Vertebral Column	328.29
Brain	1382.45
* swallowing organs from a secondary, internal dataset of 20 patients.	
